# *In Silico* Prediction of Novel Probiotic Species Limiting Pathogenic *Vibrio* Growth Using Constraint-Based Genome Scale Metabolic Modeling

**DOI:** 10.3389/fcimb.2021.752477

**Published:** 2021-09-29

**Authors:** Neelakantan Thulasi Devika, Ashok Kumar Jangam, Vinaya Kumar Katneni, Prasanna Kumar Patil, Suganya Nathamuni, Mudagandur Shashi Shekhar

**Affiliations:** Nutrition Genetics and Biotechnology Division, Indian Council of Agricultural Research-Central Institute of Brackishwater Aquaculture, Chennai, India

**Keywords:** constraint-based, genome-scale model, microbial community, probiotics, vibriosis, shrimp

## Abstract

The prevalence of bacterial diseases and the application of probiotics to prevent them is a common practice in shrimp aquaculture. A wide range of bacterial species/strains is utilized in probiotic formulations, with proven beneficial effects. However, knowledge of their role in inhibiting the growth of a specific pathogen is restricted. In this study, we employed constraint-based genome-scale metabolic modeling approach to screen and identify the beneficial bacteria capable of limiting the growth of *V. harveyi*, a common pathogen in shrimp culture. Genome-scale models were built for 194 species (including strains from the genera *Bacillus*, *Lactobacillus*, and *Lactococcus* and the pathogenic strain *V. harveyi*) to explore the metabolic potential of these strains under different nutrient conditions in a consortium. *In silico*-based phenotypic analysis on 193 paired models predicted six candidate strains with growth enhancement and pathogen suppression. Growth simulations reveal that mannitol and glucoronate environments mediate parasitic interactions in a pairwise community. Furthermore, in a mannitol environment, the shortlisted six strains were purely metabolite consumers without donating metabolites to *V. harveyi*. The production of acetate by the screened species in a paired community suggests the natural metabolic end product’s role in limiting pathogen survival. Our study employing *in silico* approach successfully predicted three novel candidate strains for probiotic applications, namely, *Bacillus* sp 1 (identified as *B. licheniformis* in this study), *Bacillus weihaiensis* Alg07, and *Lactobacillus lindneri* TMW 1.1993. The study is the first to apply genomic-scale metabolic models for aquaculture applications to detect bacterial species limiting *Vibrio harveyi* growth.

## Introduction

Microbes naturally exist as a community with a complex web of interactions that define the microbial community structure. The coordinated interactions between microbes enable metabolic processes such as cross-feeding, degradation of complex saccharides (Freilich et al., 2011), or synthesis of complex molecules (McCarty and Ledesma-Amaro, 2019). Advancement in sequencing has provided a potential resource for constructing genome-scale metabolic models (GSM) that serve as a basis to explore metabolic capabilities in microbial communities. Constraint-based modeling is a well-established approach that has been successfully applied for *in silico* prediction of microbial interactions (Henson et al., 2019; Fang et al., 2020). This approach has been applied from individual to consortium of species with the potential to discern the metabolic capabilities and interactions that operate in a microbial community (Heinken and Thiele, 2015b; Magnúsdóttir et al., 2017).

Shrimp aquaculture is among the fastest-growing farming sectors globally in the coastal regions (FAO, 2020). However, the shrimp farming suffers from frequent bacterial, fungal, and viral outbreaks (Seibert and Pinto, 2012; Alfiansah et al., 2018). According to a survey of key shrimp farming states in India, economic losses owing to shrimp infections were 0.21 million tons worth $1.02 billion in the 2018–2019 fiscal year (Patil et al., 2021). Among the bacterial pathogens, the major *Vibrio* species affecting shrimp aquaculture include *V. parahaemolyticus*, *V. vulnificus*, *V. furnissii*, *V. campbellii*, *V. harveyi*, *V. alginolyticus*, and *V. anguillarum* (Saulnier et al., 2000; Kumar et al., 2020), leading to vibriosis.

A challenging problem that arises in this domain is the possible unscientific use of antibiotics and chemotherapeutic agents leading to drug or antibiotic-resistant bacteria in aquaculture (Kang et al., 2014). In the aquaculture sector, probiotics, prebiotics, and synbiotics are widely used to improve growth performance and enhance immunity and disease resistance (Hoseinifar et al., 2018). The probiotic organisms produce natural antimicrobial compounds such as organic acids, fatty acids, hydrogen peroxide, and bacteriocins that prevent pathogenic organism’s growth and confer positive health benefits to the host (Knipe et al., 2020). The application of probiotic in shrimp farming is a key factor implicated in bioremediation, water quality improvement (Verschuere et al., 2000), enhancing the nutritional benefit and antimicrobial activity against pathogenic microorganisms (Tamilarasu et al., 2021).

Earlier studies have reported the use of beneficial bacteria for their ability to inhibit pathogen through co-culture experiments supplemented with carbohydrates or prebiotics (Fooks and Gibson, 2002; Rurangwa et al., 2009). In a study, Fierro-Coronado et al. (2018) have investigated the role of fulvic acid in improving the survival of *Litopenaeus vannamei* challenged with *Vibrio parahaemolyticus*. However, the exact molecular mechanism by which the probiotic species suppress pathogen growth remain unknown, but their role in improving the health of shrimp is well established (García-Medel et al., 2020; Wang et al., 2020; Nair et al., 2021). Moreover, screening bacterial strains for probiotic properties on a large scale *via* conventional *in vivo* and *in vitro* methods is labor-intensive, time-consuming, and expensive. Further, screening at the species level is not sufficient as the beneficial properties are strain-dependent (Campana et al., 2017; McFarland et al., 2018). Therefore, it is essential to delineate the strains limiting the survival of pathogens through *in silico* approaches. These challenges, in turn, have rendered constraint-based genome-scale modeling approach as a choice for the selection of beneficial bacterial strains by analyzing the metabolic capabilities.

We developed a strain-specific genome-scale metabolic model concentrating on *Lactobacilli*, *Bacilli*, and *Lactococci* genera having closed-genome sequences. Moreover, the species from these genera have demonstrated health advantages (Sahandi et al., 2019). The pathogenic strain *V. harveyi* employed in this study is associated with infections in shellfish, finfish, corals, and molluscs, leading to substantial economic loss to the farmed species in both brackish water and marine aquaculture (Darshanee Ruwandeepika et al., 2012). The study aims to predict the potential species limiting the growth of *V. harveyi* and analyzes the metabolic interactions and exchanges operating under different nutrient environments in the microbial communities using *in silico*-based genome-scale metabolic models. The contributions made here accelerate the screening process by identifying the candidate strains for experimental validation to evaluate their efficacy against pathogenic strains. This study represents a pioneering work in screening the species through a constraint-based approach against a shrimp pathogen.

## Methods

### Dataset

The strains belonging to the genus, namely, *Bacillus*, *Lactobacillus*, and *Lactococcus*, for which the whole genome sequences were available, were used in the present study. The genome and protein sequences corresponding to three genera comprising 193 different strains were downloaded from NCBI RefSeq (as of 11/02/2020). This includes 106 strains of *Bacillus*, 81 strains of *Lactobacillus*, and 6 strains of *Lactococcus*. The dataset also contains a pathogenic strain *V. harveyi* QT520, linked to pathogenesis in marine organism (**Supplementary Table 1**).

### Model Reconstruction

The genome-scale metabolic models were built with CarveMe v1.2.2. CarveMe generates automated models using a top-down approach for single and community species (Machado et al., 2018). CarveMe converts the universal metabolic model into an organism-specific model by removing reactions and metabolites that are unlikely to be present in the given organism. Each of the 194 protein sequences retrieved was given as an input in CarveMe to build a draft SBML model. The model statistics of 194 GSM generated is summarized in **Supplementary Table 2**. The resulting SBML models were gap filled and grown in a defined medium (**Supplementary Table 3**) in anaerobic conditions with biomass components specific to Gram-negative and Gram-positive bacteria to make the draft model functional. The individual models were merged in CarveMe using the merge community function into groups of varying sizes, and each strain was assigned its own compartment and a shared extracellular environment.

### Computing Growth Using Flux Balance Analysis

Simulations were performed with COBRA toolbox v3.0 (Heirendt et al., 2011) in Matlab R2018b using the linear programming solver *glpk*. Each microbial consortium was subjected to flux balance analysis (FBA), a constraint-based approach to predict the flow of metabolites through a metabolic network (Orth et al., 2010). FBA uses linear programming formulation and operates under steady-state conditions using stoichiometric matrix obtained from metabolic models, mathematically expressed as follows:


$$Objective:Max\cdot v_{bio}s.t.\;S.v=0Lj\leq vj\leq Uj$$

where *S* is a stoichiometric matrix of size *m*×*n*, *m* is the number of metabolites, and *n* is the number of reactions, *v* represents the flux through all reactions, *Lj* and *Uj* are the lower and upper bound flux of each reaction *j*.

Simulations were performed by maximizing the objective function, i.e., the biomass reaction, while constraining the uptake rates of amino acids and essential vitamins/nutrients to −1 mmol/gDw/h. A substrate uptake of −10 mmol/gDw/h was constrained for glucose, fructose, mannitol, galactose, mannose, N-acetyl-D-glucosamine (N-acgam), ribose, glucoronate, and arabinose, and uptake of −5 mmol/gDw/h for sucrose, maltose, cellobiose, trehalose, lactose, and maltotriose environments (**Supplementary Table 4**). All the models were simulated in an anaerobic environment by setting the lower bounds of oxygen exchange to zero. An *in silico* growth rate of at least 0.01/h was considered the organism’s ability to take up the carbon source.

### Flux Variability Analysis

Flux variability analysis (FVA) computes the flux range through each reaction while maintaining maximum biomass production (Mahadevan and Schilling, 2003).


$$Maximize,\;Minimize\;v_{j}S.t.\;S.v=0v_{j}^{\min}\leq v\geq v_{j}^{\max}$$

where *v* represents the maximum and minimum flux through each reaction *j.* Due to the long computation time, we performed FVA only on the shortlisted models to determine the production of major short-chain fatty acids (SCFA), such as acetate, lactate, ethanol, formate, succinate, and butyrate. The flux value above 1e-06 mmol/gDW/h was considered an organism’s ability to produce the metabolites while maximizing the biomass reaction.7

### Categorizing Paired Communities

We classified the strains into three categories as suggested by Heinken and Thiele (2015b), summarized as follows: (i) An increase in the growth rate of at least 10% under pairwise community compared to a single strain grown in the same environment, and unchanged growth of the pathogen in relation to a single strain. (ii) A strain with the same growth rate in paired and single in the same environment and a 10% decrease in growth for the pathogen over the single strain. (iii) A minimum of 10% increase in growth rate in paired over single in the same environment and a 10% decrease in growth of pathogen over single in same environment.

### Computing Metabolic Exchanges

Species Metabolic Interaction Analysis (SMETANA v1.2.0), a mixed-integer linear programming method, was used to compute the metabolites exchanged between species in a paired community under a defined medium (Zelezniak et al., 2015). The *smetana* command was used with the flags “–flavor cobra” with *detailed mode* using the default CPLEX solver. Among the different scores generated, we analyzed only the SMETANA score, as this potentially indicates the strength of metabolic interactions between organisms. SMETANA score varies between 0 and 1, wherein 0 represents no interaction and 1 represents certain interaction between species.

### Phylogenetic Analysis

The phylogenetic analysis was performed with MEGA7.0 software using the 16S rRNA genes of shortlisted species to understand their phylogenetic relationship (Kumar et al., 2016). We used MEGA software’s ClustalW program for multiple sequence alignment with bootstrap set to 1,000 using the maximum likelihood method to generate phylogenetic trees.

### Average Nucleotide Identity

The average nucleotide identity (ANI) indices were computed using the Python library pyANI v0.20 to study genome level similarities among the selected species (Pritchard, 2015). PyANI uses Mummer or Blast to compute similarity indices. ANI was run with the argument “ANIm”, which calculates indexes with Mummer.

### Statistical Analysis

The growth values derived from FBA were subjected to non-parametric one-way ANOVA with Kruskal-Wallis test using the R package for all combinations of shortlisted six species across four different media. The data visualization was performed with ggplot2.

## Results

### Screening Potential Species Based on Growth Benefit and Suppression of Pathogen

As an initial core analysis, each model was subject to FBA in an anaerobic media with single carbon source (15 different carbon sources were used). As shown in **Supplementary Figure 1**, the single models of 193 strains exhibited a wide range of simulated growth rates in each of the 15 nutrient environments. About carbon source utilization, 32 strains comprising 20 *Bacilli*, 11 *Lactobacilli*, and 1 *Lactococci* showed fermentation in all 15 environments. All 193 individual models showed *in silico* growth in maltose and glucose environments. Most strains (40–70%) fermented the carbon sources glucoronate, arabinose, and galactose. The strains *L.kunkeei* MP2 and *L.* sp. BHWM 4 are the least fermenting ones that utilized only five carbon sources. Additionally, phenotypic prediction on the pathogenic strain *V. harveyi* revealed no *in silico* growth under arabinose (**Supplementary Figure 2**).

**Figure 1:**
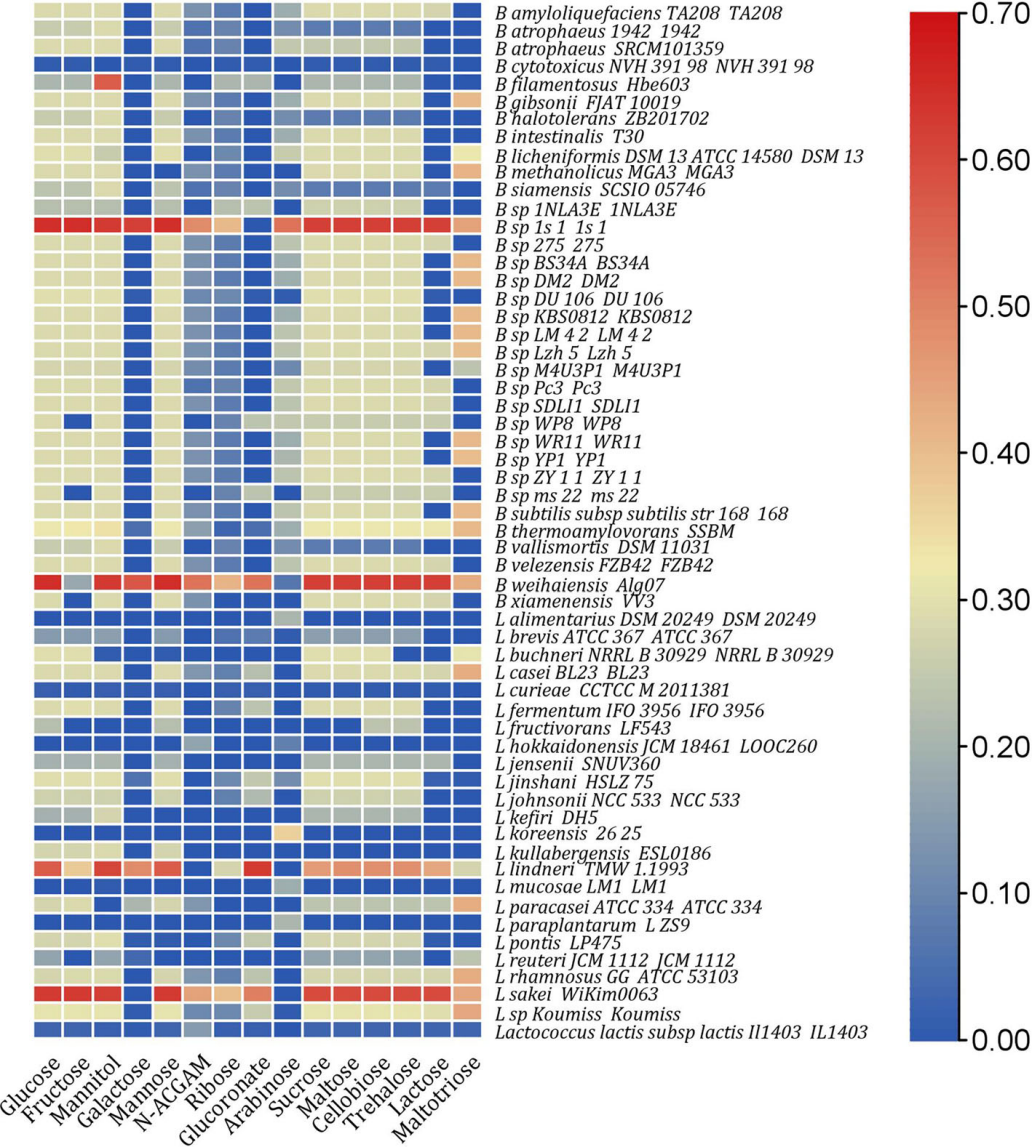
Heat map representing computed growth rates of strains with significant increase in growth rates (at least 10% uplift in *in silico* growth rate than single species) in a paired community with 15 different nutrient environments.

Next, the single 193 models were merged with the pathogenic strain *V. harveyi* to perform pairwise community simulations. Only 48% of the strains in a paired community showed *in silico* growth, with only 30% showing a 10% increase in growth compared to single strains under diverse nutrient environments, as shown in **Figure 1**. Moreover, only 30% of strains in the paired community grew on the sole carbon sources, namely, glucose and maltose, compared to the single strains, where all 193 strains grew. In a paired community, three strains, namely, *B. thermoamylovorans* SSBM, *B. weihaiensis* Alg07, and *L.* sp. *Koumiss*, could survive across all nutrient environments.

In paired communities, *V. harveyi* could grow with 186 out of 193 strains, as shown in **Supplementary Figure 3**, on 15 different nutrient environments. Under galactose environment, *V. harveyi* showed significant growth *in silico*. It was noted that only nine strains, namely, *Bacillus sp* 1s 1, *B. thermoamylovorans* SSBM, *B. weihaiensis* Alg07, *L. jinshani* HSLZ 75, *L. lindneri* TMW 1.1993, *L. paracasei* ATCC 334, *L. pontis* LP475, *L. reuteri* JCM 1112, and *L. sp Koumiss*, limited the growth of the pathogenic strain under galactose environment. Additionally, growth simulation revealed a limited growth for *V. harveyi* under mannitol environment in a paired community.

### Mannitol Exhibits Parasitic Interaction in Pairwise Community Models

Based on the *in silico* phenotypic analysis, we categorized the strains as tabulated in **Supplementary Table 5**. We focussed our analysis on the paired models where an increase in growth rate was observed for the strains while a limited *in silico* growth rate for pathogen. A total of 48 strains, comprising 30 bacilli and 18 lactobacilli, fall into this category.

**Figure 2:**
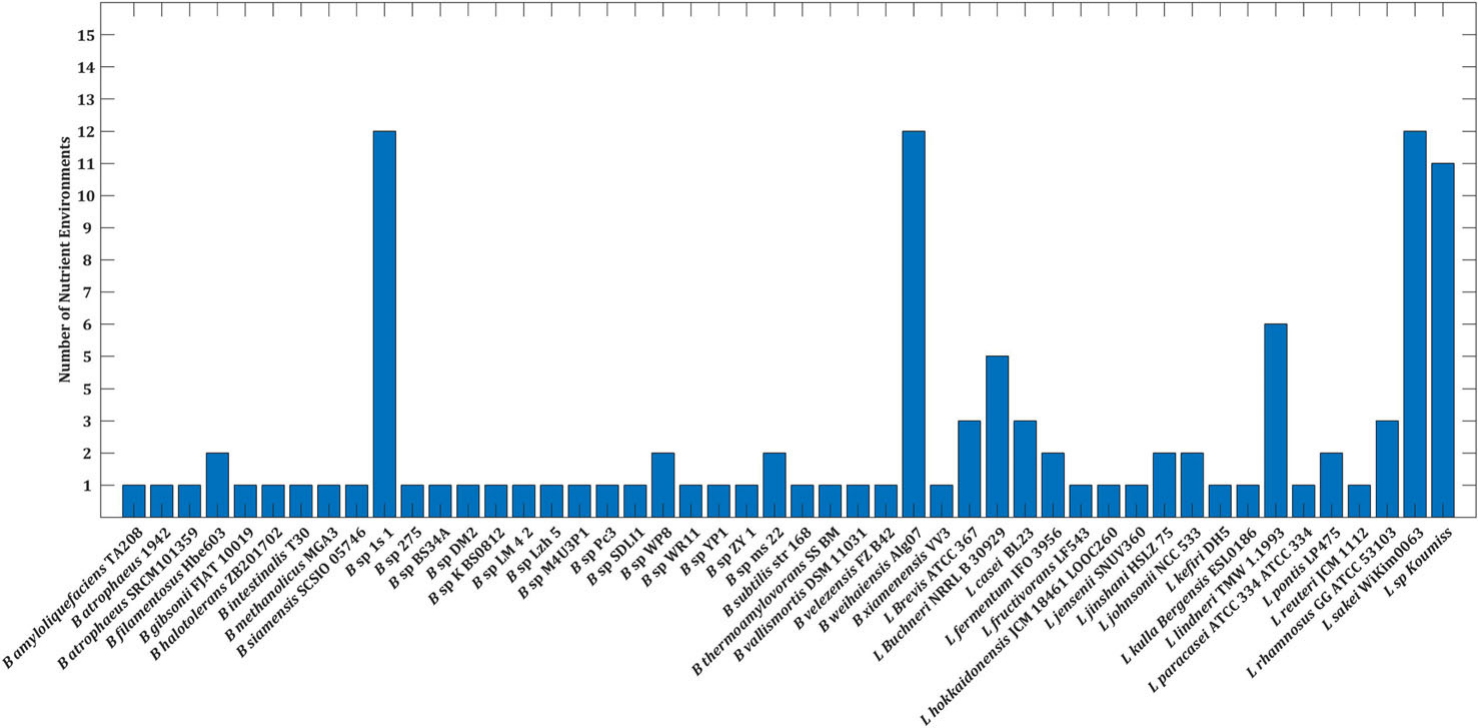
Barplot depicting the number of nutrient environments in which strains limits growth of *V. harveyi*.

Prior work by Heinken and Thiele (2015a) has classified microbial communities into six types of interactions based on growth rates: parasitism, amensalism, commensalism, mutualism, neutralism, and competition. As the study’s goal was to identify the strains that limit the growth of the pathogen as well as enhance the growth of the strains, we were interested in parasitic interaction. Our analysis revealed that only 15% of the strains accounted for parasitic interactions (**Supplementary Figure 4**). The six strains, namely, *Bacillus* sp 1s 1, *B. weihaiensis* Alg07, *L. sakei* WiKim0063, *L. sp Koumiss*, *L. lindneri* TMW 1.1993, and *L. buchneri* NRRL B 30929, which showed parasitic interaction, also outperformed the other species in at least five nutrient environments, both improving growth and limiting pathogen survival (**Figure 2**). The different interaction types exhibited by 48 strains are tabulated in **Supplementary Table 6**.

Out of the 15 nutrient environments used in this study, pathogen survival was limited majorly in the mannitol environment, as shown in **Supplementary Figure 5**. Analysis of the 48 strains reveals metabolic gains on four species, namely, *B. thermoamylovorans* SSBM, *B. weihaiensis* Alg07, *L. jinshani* HSLZ 75, and *L. lindneri* TMW 1.1993. Among the four species, the highest metabolic gain is achieved by *L. lindneri* TMW 1.1993, upon paired with the pathogen (**Supplementary Figure 6**).

### Cross-Fed Metabolites in Pairwise Community

SMETANA identified 87 metabolites cross-fed across the pairwise community for the 48 filtered species (**Supplementary Table 7**). Among the metabolites predicted, minerals and amino acids were frequently exchanged. In addition, lactate, acetate, ethanol, fumarate, and succinate have also been predicted to be exchanged. Out of the 87 pan metabolites predicted *in silico*, 58 metabolites were environment independent, with exchange occurring in all nutrient environments.

**Figure 3:**
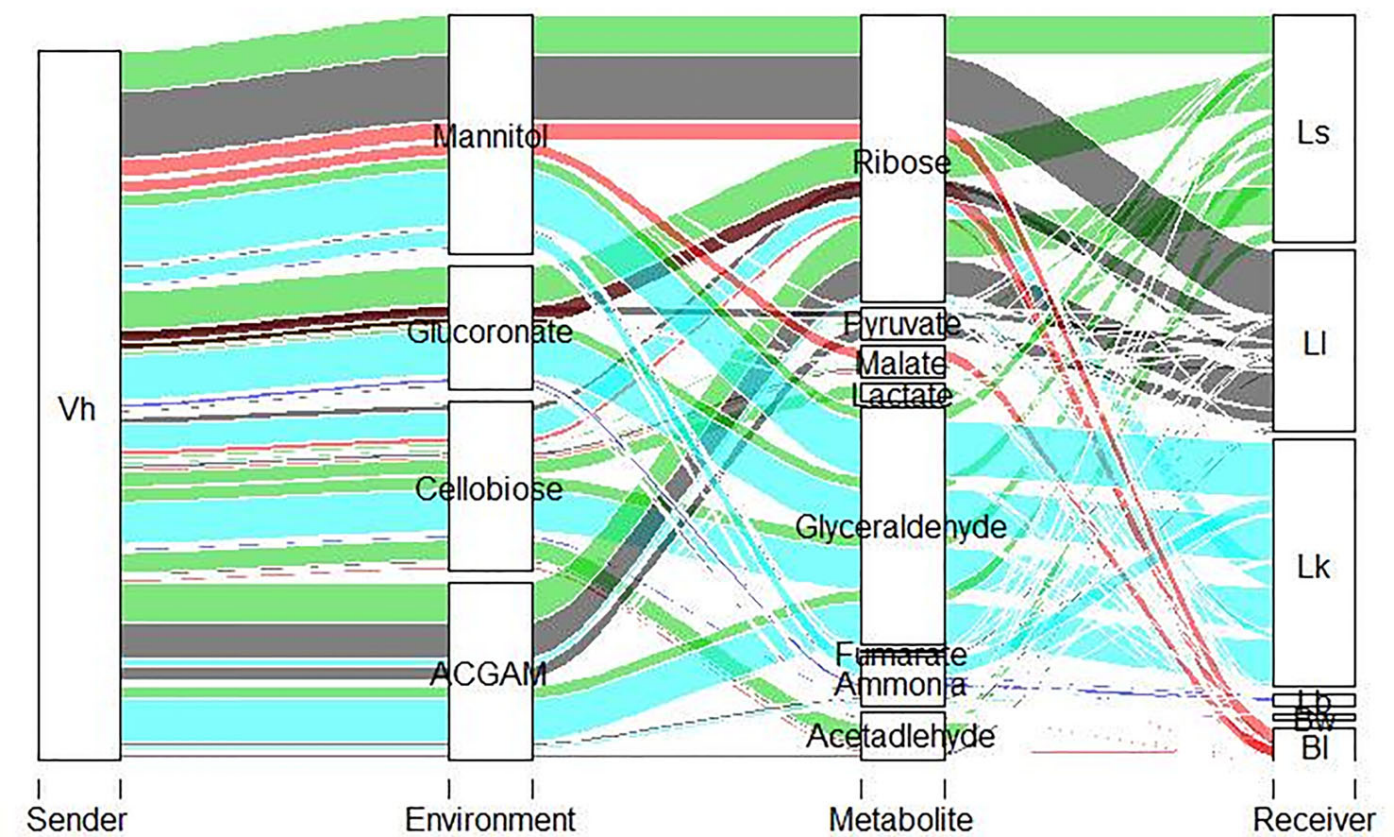
Alluvial plot depicting top metabolites exchanged between shortlisted species and *V. harveyi* with thickness of the line denoting the SMETANA scores, which range between 0 and 1.

Moreover, out of the 48 strains, eight strains, namely, *L. fermentum* IFO 3956, *L. hokkaidonensis* JCM 18461 LOOC260, *L. kullabergensis* ESL0186, *L. lindneri* TMW 1.1993, *L. pontis* LP475, *L. reuteri* JCM 1112, *L. sakei* WiKim0063, and *L.* sp *Koumiss*, were only consumers, i.e., receiving metabolites from the pathogen based on our *in silico* predictions. All these strains have shown the ability to benefit from pathogen across all the nutrient environments by consuming metabolites. Among the metabolites cross-fed in the shortlisted strains (**Figure 3**), *L. lindneri* TMW 1.1993 achieved significant *in silico* growth benefits by uptaking carbon sources, namely, glucose and ribose, predicted to be secreted from the pathogen.

### Short-Chain Fatty Acids Profile Across Nutrient Environments

The flux profiles, i.e., the qualitative increase or decrease of SCFA, were inferred using FVA in single and paired models to contextualize the pairwise community models towards production of metabolites across environments. When comparing pairwise communities to single species, acetate and ethanol fluxes increased. The lactate production potential of 48 species varied significantly, with single models exhibiting higher flux than pairwise community models. Similarly, the ability of the organism to produce succinate and fumarate was higher in single species. All 48 paired species were unable to produce butyrate. A total of 10 species, as listed in **Supplementary Table 8**, could synthesize five of the six metabolites in at least one of the nutritional conditions.

### Community Simulation With Potential Species

To unravel how the interactions among shortlisted species limit the growth of *V. harveyi*, we made merged models from all possible combinations of six strains for generating different consortia and subjected to FBA in four different nutrient environments. The four nutrients, namely, mannitol, N-acgam, glucoronate, and cellobiose, were chosen based on strain survival and limiting pathogen growth in these environments. The *in silico* growth rate listed in **Supplementary Table 9** is the sum of the biomass reactions of the potential strains under each environment in different consortia. The results indicate that the maximum simulated growth rate of strains under mannitol, N-acgam, glucoronate, and cellobiose environment were 0.17, 0.14, 0.17, and 0.16/h, respectively. The consortium consisting of *Bacillus* sp 1s, *B. weihaiensis* Alg07, and *L. lindneri* TMW 1.1993 was the most effective combinations in growth enhancement and limiting pathogen survival.

**Figure 4:**
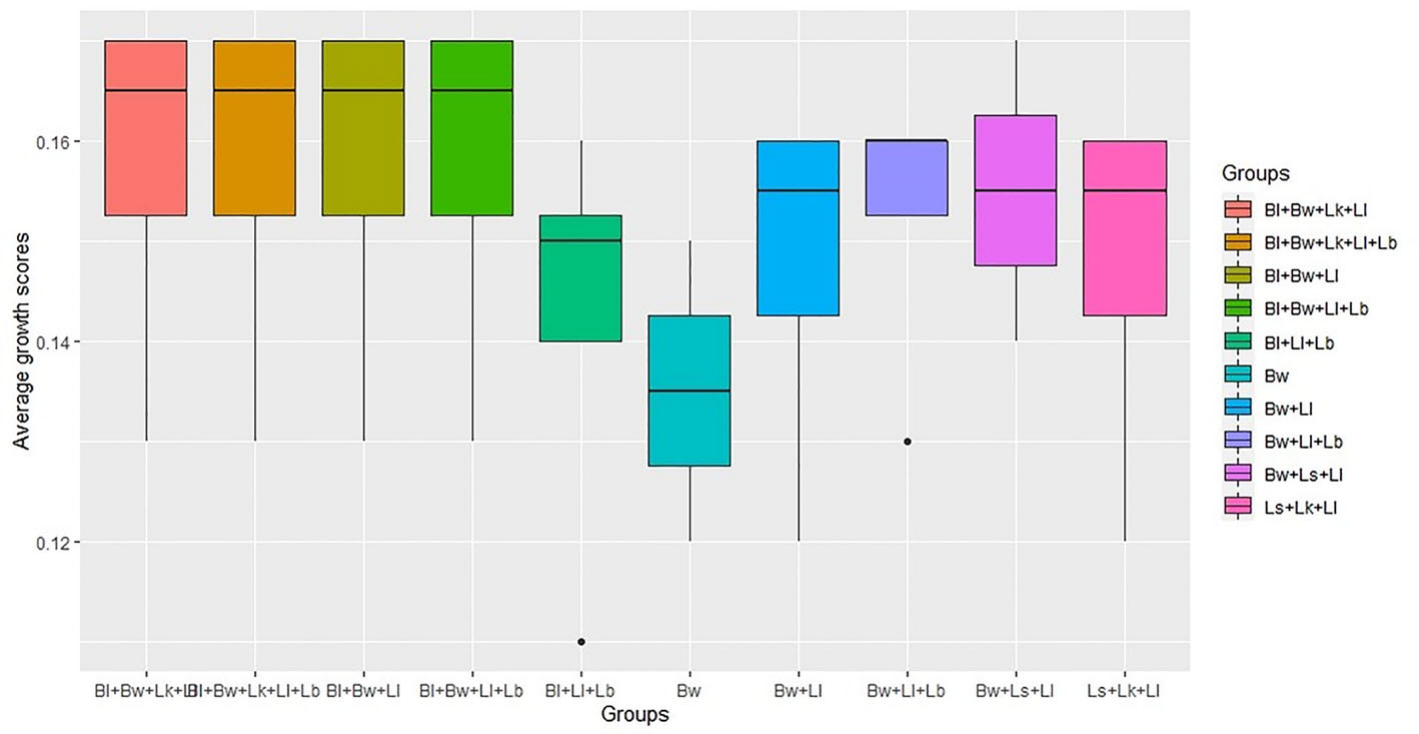
Top 10 community model groups with highest average growth scores (Bw, *B. weihaiensis*; Ll, *L. lindneri*; Bl, *B. licheniformis*; Ls, *L. sakei*; Lk, *L. Koumiss*; Lb, *L. buchneri*).

Non-parametric one-way ANOVA revealed a significant difference between the groups tested in community simulations. The test resulted in Kruskal-Wallis chi-square value of 10.10 at p-value <0.05, indicating the significance between the species combinations. The top 10 groups that exhibited the highest average scores are depicted in **Figure 4**. Among the total combinations, *B. licheniformis*, *B. weihaiensis*, and *L. lindneri* were the best combinations that exhibited the highest *in silico* growth rates in the presence of *V. harveyi.*

## Discussion

This study reports the constraint-based metabolic modelling approach applied for the first time in the aquaculture ecosystem to screen potential strains limiting the growth of the pathogen. The individual models constructed from the genome of 193 strains exhibited better i*n silico* growth in over 15 different nutrient environments. In contrast, limited *in silico* growth was observed with the paired models. The *in silico* growth impediment of strains observed in the paired models attributes to the metabolites derived from *V. harveyi*, a known property of the *Vibrio* spp. (Balakrishnan et al., 2014).

We identified 48 out of 193 strains through a flux-based approach to be ideal candidates limiting the growth of *V. harveyi* in specific nutrient environments. It is important to note that many strains out of these 48, like *B. subtilis*, *L. casei*, *L. rhamnosus*, *L. paracasei*, *L. sakei*, *L. sp Koumiss*, and *L. buchneri*, have already reported to be probiotic species (Lim et al., 2014; Hill et al., 2018; Westerik et al., 2018; Rhayat et al., 2019; Xu et al., 2019; García-Medel et al., 2020; Liao et al., 2020; Tang et al., 2020). Flux variability analysis revealed the production of SCFA, mainly acetate, by a majority of the strains in paired models, signifying the role of this natural by-product towards growth inhibition of the pathogen. Earlier, Mine and Boopathy (2011) also reported the role of organic acids such as formic acid, acetic acid, propionic acid, and butyric acid in inhibiting the *V. harveyi*.

The constraint-based approach employed in the current study screened several *Lactobacilli* strains as potential candidates limiting *V. harveyi*. The shortlisted strain *L. buchneri* NRRL B 30929 was originally isolated from an ethanol fermentation plant which has broad substrate utilization capability (Heinl and Grabherr, 2017).

In our *in silico* analysis, *L. buchneri* exhibited parasitic interaction limiting the pathogen surivival. Another shortlisted species, *L. sakei*, isolated from fish and meat products (Lim et al., 2014), reduce the count of *Vibrio* species in different live prey such as *Artemia franciscana*, *Brachionus plicatilis*, and *Tigriopus japonicas* (Sahandi et al., 2019). It has been reported that *L. sakei* showed a stronger killing effect on pathogenic *V. parahaemolyticus*, making it a promising candidate in controlling vibriosis (Le and Yang, 2018). The *in silico* analysis presented in this study revealed that *L. sakei* strain benefited from metabolites generated from *V. harveyi*, exhibiting a parasitic relationship by experiencing growth benefit.

*L. lindneri* is one of the novel *Lactobacillus* strains identified through constraint-based approach as a potential candidate against *V. harveyi* in our study. Interestingly, *L. lindneri* is associated with the following metabolic capabilities: (i) Flux-based Analysis revealed the ability to limit V*. harveyi* in many of the nutrient environments. (ii) Metabolic gain achieved by *L. lindneri*, which signifies a species’ ability to utilize a nutrient in a paired model where the same nutrient cannot be catabolized alone by the species. (iii) SMETANA-based analysis reveal *L. lindneri* as the consumer of metabolites donated by *V.harveyi.* In addition to the distinguished metabolic capabilities exhibited by *L. lindneri* in simulations performed in this study, it has also exhibited a close phylogenetic affiliation to known probiotic strains. The 16s rRNA-based phylogeny with a bootstrap value of 93% places greater confidence that *L. lindneri* is closely related to *L. fructivorans* (**Supplementary Figure 7**). The strain *L. fructivorans* is previously reported to significantly decrease larval mortality in sea bream (Carnevali et al., 2004).

Among the *Bacillus* strains shortlisted in this study, *B. weihaiensis* Alg07 exhibited parasitic interactions in the pairwise community. The *B. weihaiensis* Alg07 strain is associated with polysaccharide degradation and promotes the nutrient cycle and other essential functions in the marine ecosystem (Zhu et al., 2016). Another shortlisted strain, *Bacillus* sp 1s, exhibited proximity with *B. licheniformis* DSM 13 ATCC 14580 using ANI analysis (**Supplementary Table 10**). Several extracellular bio-compounds, including vitamins and enzymes, are produced by *B. licheniformis*, improving nutrient digestion and innate immunity (Tachibana et al., 2021). Moreover, the probiotic effect of *B. licheniformis* BCR 4-3 has been reported to increase resistance in *Litopenaeus vannamei* challenged with *V. parahaemolyticus* (García-Medel et al., 2020). In another study, the hemolytic activity of *V. harveyi* has been reported to be reduced by *B. licheniformis* (Nakayama et al., 2009).

In a microbial community, the nutrient environment significantly influences the interactions between species (Freilich et al., 2011). Our study highlighted the role of sugar alcohol mannitol, which retained its influence on growth benefit on beneficial species and limiting pathogen in different communities generated. The growth improvement effect of mannitol on *Lactobacillus* reported by Liong and Shah (2005) and limiting effect on *V. harveyi* observed in our study indicate the role of this sugar alcohol in the parasitic interactions. Simulations on multiple consortia generated with all possible combinations of potential strains revealed the community comprising *Bacillus* sp 1s, *B. weihaiensi*s Alg07, and *L. lindneri* TMW 1.1993 to be the ideal combination in reducing the pathogen’s survival in the studied nutrient environments. This study also demonstrated the potential of genome-scale model to identify the ideal consortia of compatible potential strains for further testing and applications in the field.

The present study was conducted with the automated genome-scale model generated due to the non-availability of readily available manually curated published models. In addition, the scope of the study is limited to monosaccharide and disaccharide nutrient environments only. Future works with similar methods as followed in the study could provide further insights into the metabolic interactions between the probiotic and the pathogen if curated models with diverse nutrient environments are used and validated through experiments.

## Conclusion

Large-scale screening for identifying beneficial microbes using experimental methods is difficult and time-consuming. However, the genome-scale modeling work presented here provides a feasible, effective, and alternative approach to accelerate the screening of larger microbial communities and shorten the path to experiments. Through the metabolic modelling approach, we can narrow down the search of potential probiotic candidates from the huge pool of species with closed genome availability, reducing experimental efforts and saving time and resources. The study also suggests the importance of nutrient environments in driving the parasitic interactions facilitating the growth of beneficial microbes. Moreover, with the exchange of metabolites observed in the paired models, the beneficial bacteria might gain an advantage and inhibit pathogen growth. Collectively, this work illustrated the importance of constraint-based genome-scale modeling to shortlist potential strains that could go for experimental validation.

## Data Availability Statement

The original contributions presented in the study are included in the article/**Supplementary Material**. Further inquiries can be directed to the corresponding author.

## Author Contributions

ND performed the study and analyzed the data and prepared the original draft of the manuscript. AJ, VK, and PP conceived, designed, and analyzed the study and read, revised, and approved the manuscript. SN performed bioinformatics analysis, and MS revised the manuscript. All authors contributed to the article and approved the submitted version.

## Conflict of Interest

The authors declare that the research was conducted in the absence of any commercial or financial relationships that could be construed as a potential conflict of interest.

## Publisher’s Note

All claims expressed in this article are solely those of the authors and do not necessarily represent those of their affiliated organizations, or those of the publisher, the editors and the reviewers. Any product that may be evaluated in this article, or claim that may be made by its manufacturer, is not guaranteed or endorsed by the publisher.

## Supplementary Material

Supplementary Figure 1
*In silico* growth rate of 193 species on 15 different nutrient environments.

Supplementary Figure 2
*In silico* growth rate of *Vibrio harveyi* on 15 different nutrient environments.

Supplementary Figure 3 Heat map representing the *in silico* growth of *V. harveyi* QT520 in a pairwise community across 15 different nutrient environments.

Supplementary Figure 4 Interaction types exhibited by 48 species under 15 different nutrient environments. Red represents the maximum number of environments in which organisms present different types of interaction.

Supplementary Figure 5
*In silico* nutrient uptake by screened 48 species in pairwise community models.

Supplementary Figure 6 Barplot representing metabolic gains achieved by single and paired species.

Supplementary Figure 7 Phylogenetic tree of 16S rRNA gene of screened species generated using maximum likelihood method in MEGA 7.0 with bootstrap set to 1,000.
