# Supplementary material for: In Silico Prediction of Novel Probiotic Species Limiting Pathogenic Vibrio Growth Using Constraint-Based Genome Scale Metabolic Modeling
Source: Front Cell Infect Microbiol. 2021 Sep 29;11:752477. doi: 10.3389/fcimb.2021.752477 (PMC8512700; doi:10.3389/fcimb.2021.752477)
Supplement: Supplementary Figure 1 — In silico growth rate of 193 species on 15 different nutrient environments. [file Table_1.docx]

**Supplementary Figures**

**
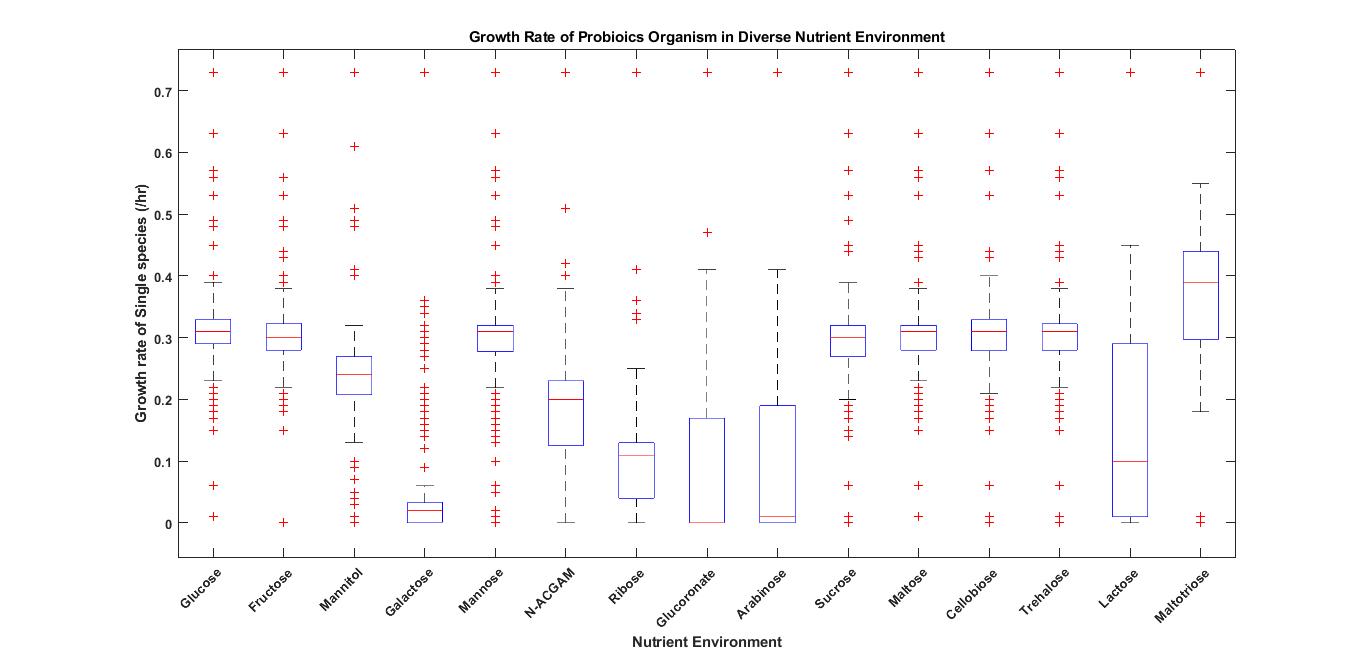
 Supplementary Figure 1**: *In silico* growth rate of 193 Species on 15 different carbon environments

**Supplementary Figure 2**: *In silico* growth rate of *Vibrio harveyi* on 15 different carbon environments

**
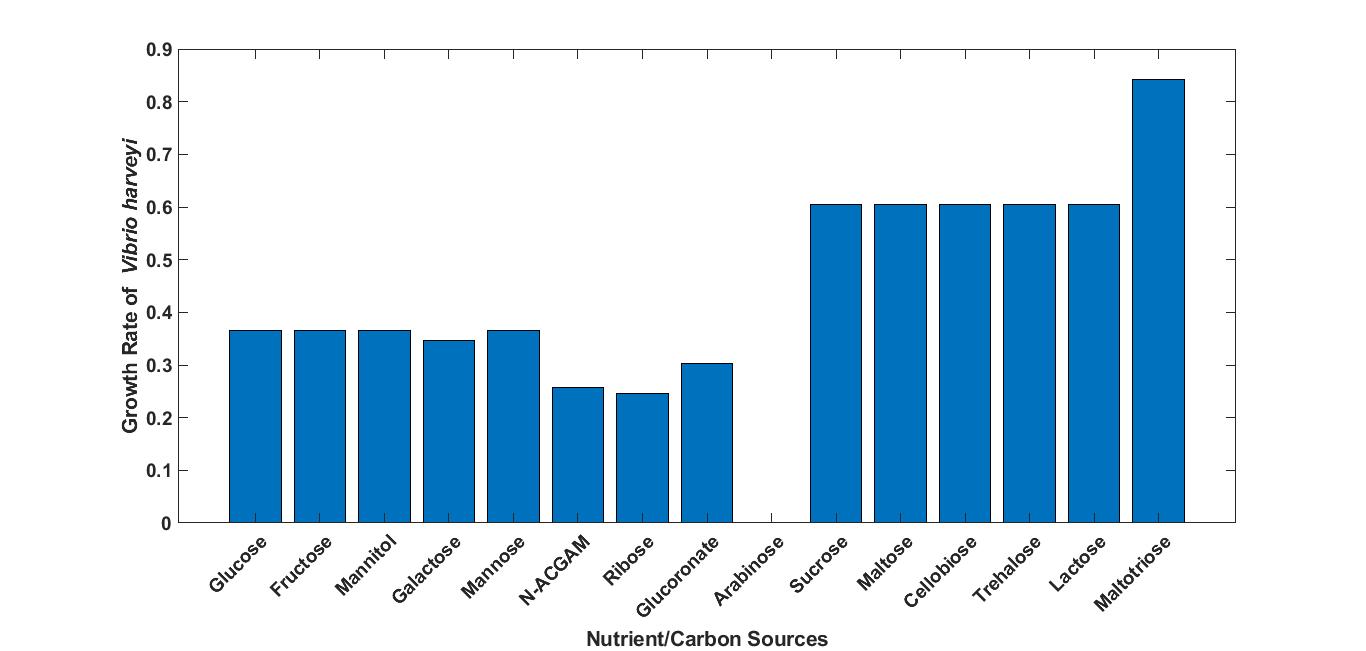
**

**Supplementary Figure 3**: Heat map representing the *in silico* growth of *V. harveyi* QT520 in a pairwise community across 15 different environments.


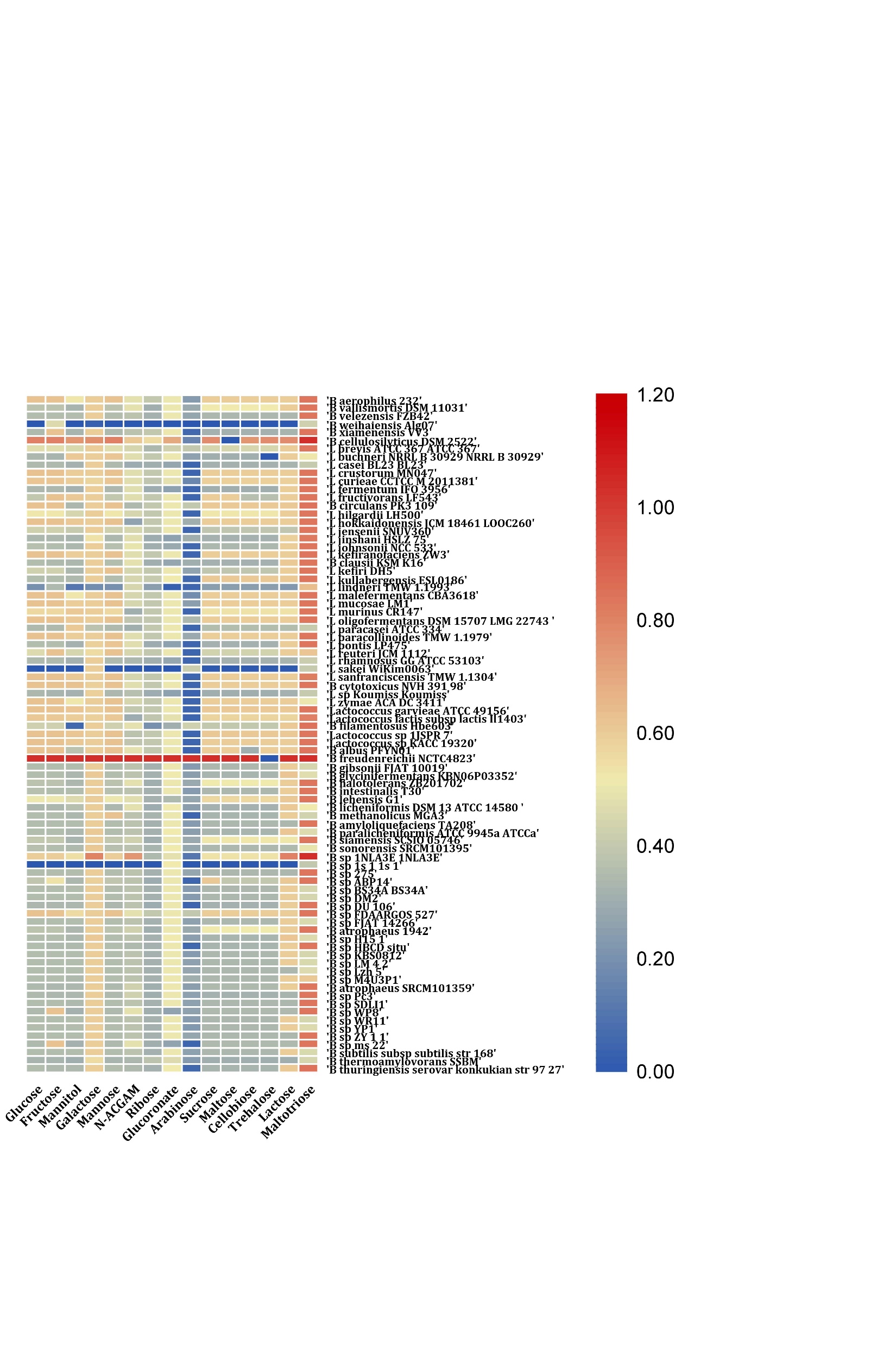


**Supplementary Figure 4:** Interaction types exhibited by 48 Species under 15 different nutrient environments. Red represents the maximum number of environments in which organism present different types of interaction.


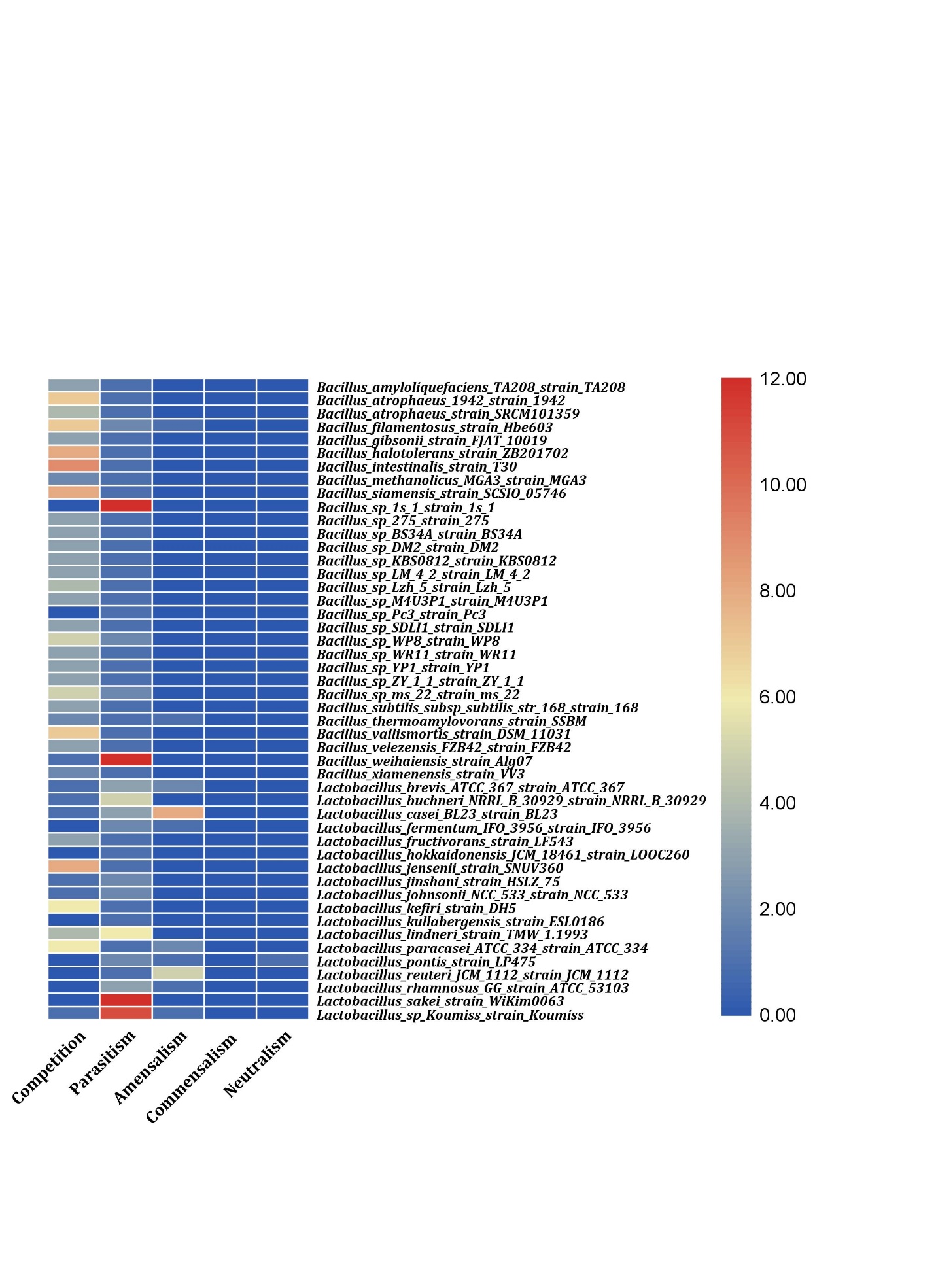


**Supplementary Figure 5**: *In silico* nutrient uptake by screened 48 species in pairwise community models.


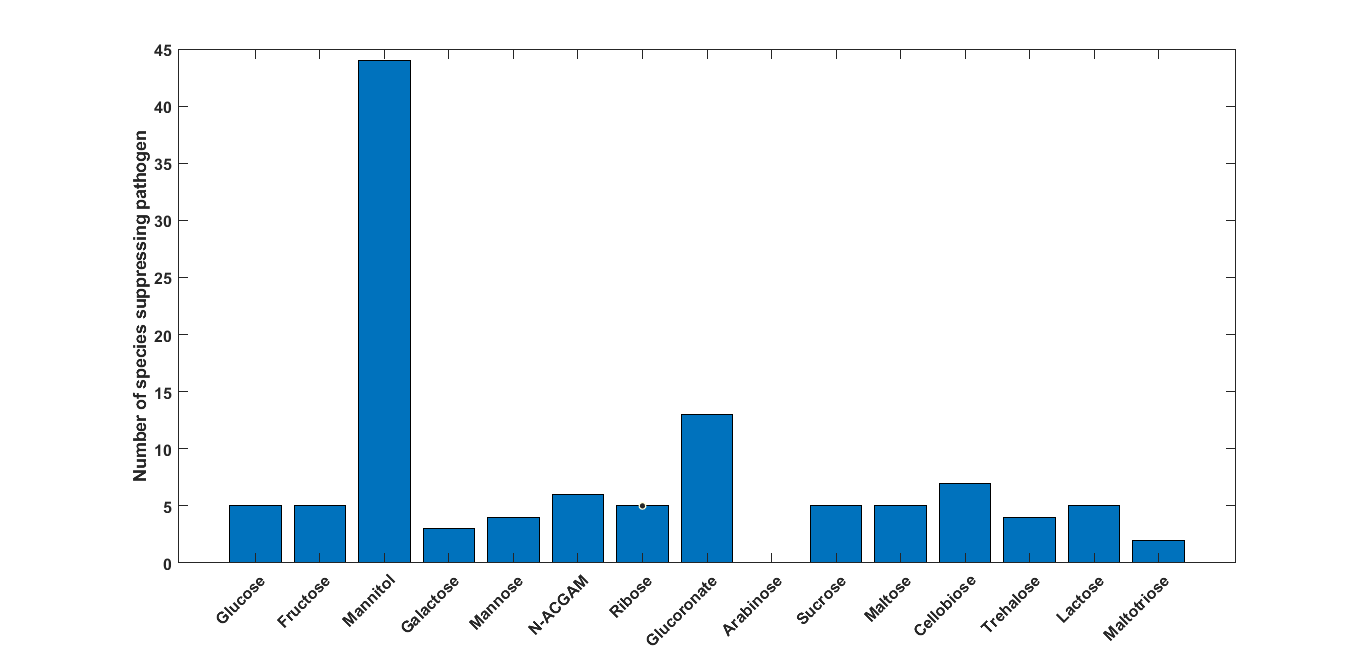


**Supplementary Figure 6**: Barplot representing metabolic gains achieved by single and paired species

**
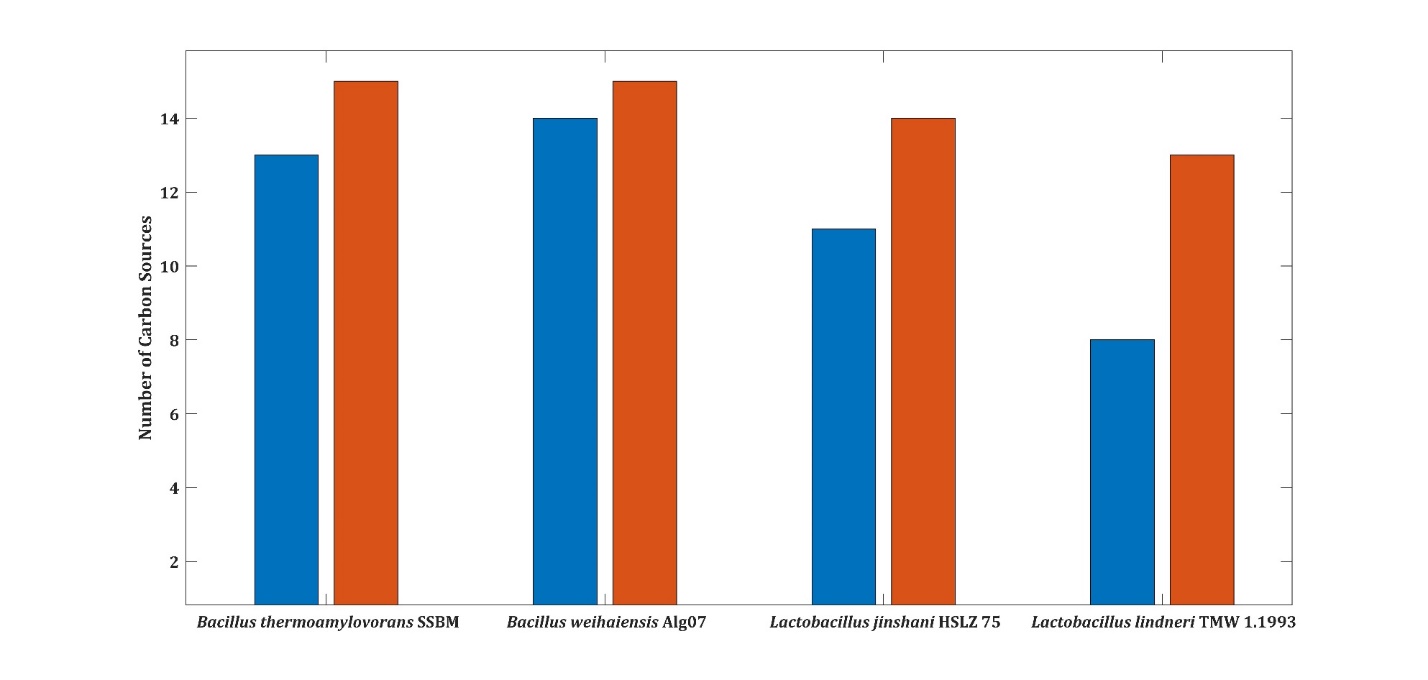
**

**
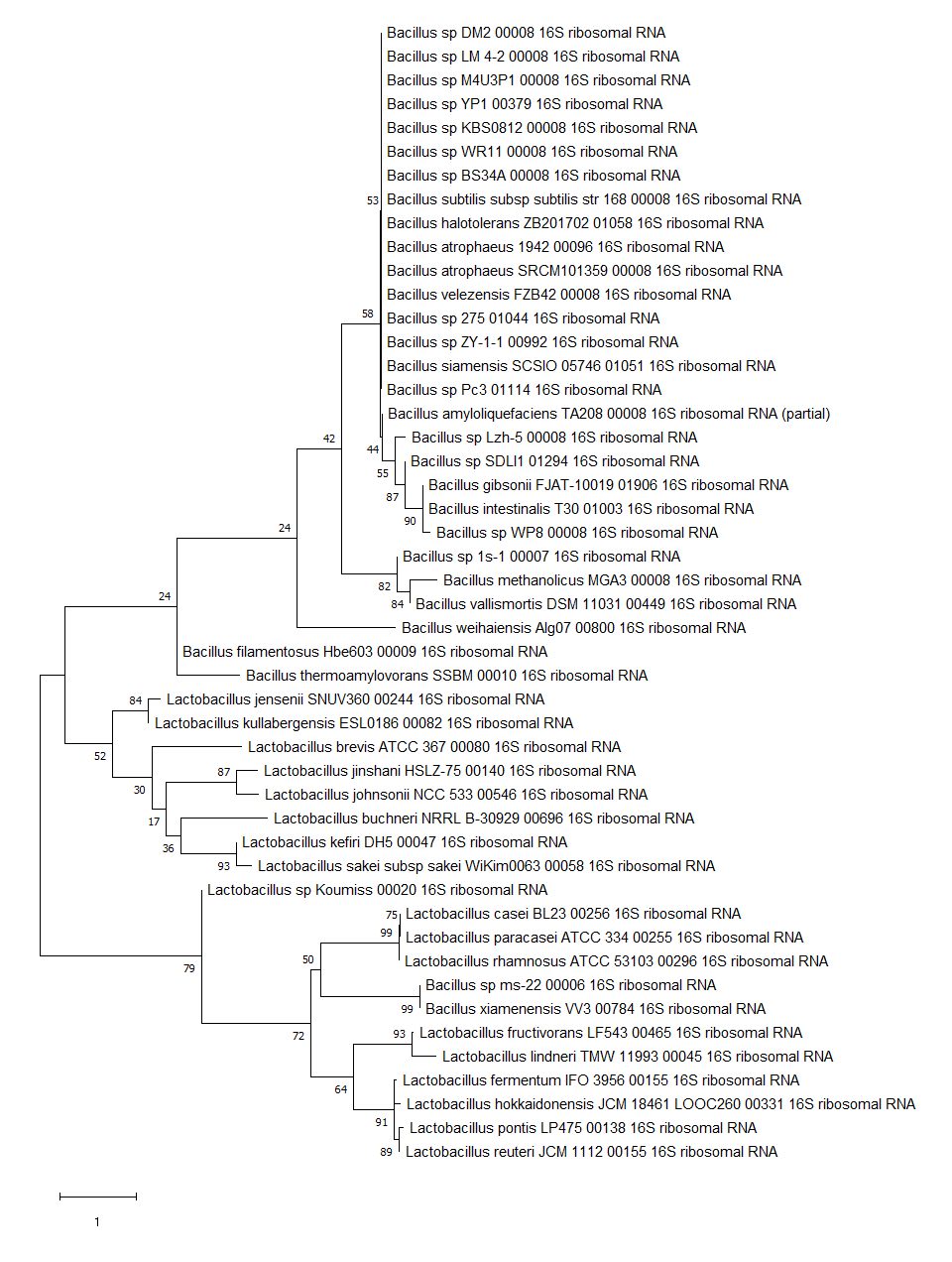
Supplementary Figure 7**: Phylogenetic tree of 16S rRNA gene of screened species generated using maximum likelihood method in MEGA 7.0 with bootstrap set to 1000
